# *Orthopox simiae* muscle abscess

**DOI:** 10.1007/s15010-022-01961-1

**Published:** 2022-12-06

**Authors:** Thomas Theo Brehm, Lennart Hermanussen, Stefan Schmiedel

**Affiliations:** 1 Section of Infectious Diseases, I. Department of Internal Medicine, University Medical Center Hamburg-Eppendorf, Martinistraße 52, 20246 Hamburg, Germany; 2 German Center for Infection Research (DZIF), Partner Site Hamburg-Lübeck-Borstel-Riems, Hamburg, Germany

A 31-year-old man presented with disseminated papular and ulcerative lesions on the face and body with few genital and anal lesions. He reported that the rash had started a few days prior on his nose. *Orthopox simiae* DNA was detected by PCR in the lesions, the throat, and the blood of the patient. He was known to be HIV-positive with a CD4 count of 30/µl due to combination antiretroviral therapy (cART) non-adherence. He denied any sexual contact but reported that he had to repeatedly share his bed with other residents of the refugee shelter where he lived. Given his poor immune status, the high number of lesions, and *Orthopox simiae* viremia, he was treated with oral tecovirimat for 14 days [1], and discharged home in a clinically improved condition. Few days later he was readmitted with strong pain and swelling in his right leg and popliteal and inguinal lymphadenopathy. T2-weighted magnetic resonance imaging (MRI) showed edema of the gastrocnemius muscle with several fluid collections with peripheral contrast enhancement compatible with infectious myositis with abscess formation. Open muscle biopsy and abscess drainage were performed, tissue samples showed chronic inflammation and tested positive for *Orthopox simiae* by PCR at a concentration of 10^8^copies/ml. Bacterial culture, mycobacterial culture, pan-bacterial and pan-fungal PCRs, as well as *Mycobacterium tuberculosis* PCR were negative. He was again treated with oral tecovirimat for 14 days and his overall clinical condition as well as the pain and swelling of the leg gradually improved over the course of the hospitalization. We show that in immunocompromised patients *Orthopox simiae* can cause infectious myositis and muscle abscesses. This is an important differential diagnosis for secondary bacterial superinfection (Fig. 1).

**Fig. 1:**
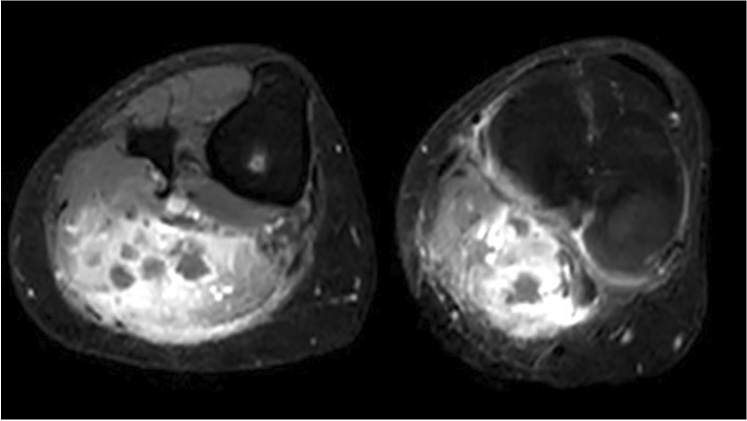
T2-weighted magnetic resonance imaging (MRI) showing edema of the gastrocnemius muscle with fluid collections and peripheral contrast enhancement. Images: Department of Diagnostic and Interventional Radiology, University Hospital Hamburg Eppendorf

## Data Availability

Not applicable.
